# Age-related differences in auditory evoked potentials as a function of task modulation during speech–nonspeech processing

**DOI:** 10.1002/brb3.188

**Published:** 2013-10-31

**Authors:** Katharina Simone Rufener, Franziskus Liem, Martin Meyer

**Affiliations:** 1 Neuroplasticity and Learning in the Normal Aging Brain (HAB LAB), University of ZurichZurich, Switzerland; 2 International Normal Aging and Plasticity Imaging Center (INAPIC), University of ZurichZurich, Switzerland; 3 Departement of Neuropsychology, University of ZurichZurich, Switzerland; 4 University Research Priority Program “Dynamics of Healthy Aging”, University of ZurichZurich, Switzerland

**Keywords:** Aging, attention, EEG, N1, P2, speech

## Abstract

**Background:**

Healthy aging is typically associated with impairment in various cognitive abilities such as memory, selective attention or executive functions. Less well observed is the fact that also language functions in general and speech processing in particular seems to be affected by age. This impairment is partly caused by pathologies of the peripheral auditory nervous system and central auditory decline and in some part also by a cognitive decay.

**Aims:**

This cross-sectional electroencephalography (EEG) study investigates temporally early electrophysiological correlates of auditory related selective attention in young (20–32 years) and older (60–74 years) healthy adults.

**Material and methods:**

In two independent tasks, we systematically modulate the subjects' focus of attention by presenting words and pseudowords as targets and white noise stimuli as distractors.

**Results:**

Behavioral data showed no difference in task accuracy between the two age samples irrespective of the modulation of attention. However, our work is the first to show that the N1-and the P2 component evoked by speech and nonspeech stimuli are specifically modulated in older adults and young adults depending on the subjects' focus of attention.

**Conclusion:**

This finding is particularly interesting in that the age-related differences in AEPs may be reflecting levels of processing that are not mirrored by the behavioral measurements.

## Introduction

Successfully taking part in everyday life requires the listener to focus his or her attention on the acoustic stream of the relevant interlocutor. Other, irrelevant information such as utterances of other speakers or background noise have to be ignored. Although a rather unspectacular situation we hardly think about in everyday life, this task demands an extensive amount of cognitive effort, specifically in attention.

Selective attention requires the ability to focus on relevant information and to ignore irrelevant information (Melara et al. 2002; Tong and Melara 2007). The ability to inhibit irrelevant information has been proposed to be the main source of age-related cognitive change (Hasher and Zacks 1988; Park et al. 1989). According to Hasher and colleagues' “Inhibitory Deficit Theory,” less inhibitory processes lead to higher requirements on working memory because more information has to be maintained in working memory. This, in turn, leads to poorer encoding of new incoming information and in consequence impaired performance. Although age-related distraction by irrelevant information has been extensively demonstrated in the visual modality (Posner and Driver 1992), studies examining auditory paradigms revealed a mixed pattern of results. This heterogeneity results in some part from the relatively small number of conducted studies. However, the major part is explained by the huge diversity of used paradigms and auditory stimuli (Guerreiro et al. 2010).

Electroencephalogram (EEG) and scalp-recorded event-related brain potentials (ERPs) are established methods in the field of cognitive neuroscience. This measurement enables researchers to gain an objective measure of neural activation patterns released by the activation of a sum of tens of thousands of synchronous firing neural cells. Moreover, this approach convinces with an excellent temporal resolution in the range of milliseconds. Therefore, ERPs are sensitive measures of the temporal dynamics and the intensity of stimulus-induced electrocortical activity during information processing (Mueller et al. 2008). These factors make EEG the method of choice when focusing on very transient patterns as can be found in speech processing and attention modulation.

The most prominent auditory evoked potential (AEP) components in the context of auditory cognition are N1 and P2, with peak amplitudes at about 100 ms and 200 ms after stimulus onset, respectively. These components are associated with early attention and orienting processes, as well as cortical arousal response (Näätänen and Picton 1987).

Previous studies on age-related differences in the waveform of auditory evoked N1 and P2 components during selective attention tasks have shown inconsistent findings. Whereas several studies indicated an enhanced N1 peak amplitude in older adults compared to younger adults (Amenedo and Diaz 1999), others do not find such differences (Brown et al. 1983; Picton et al. 1984; Barrett et al. 1987; Woods 1992; Iragui et al. 1993). The same inconsistency can be found concerning the P2 component. Whereas some authors found increased peak amplitudes in older adults (Pfefferbaum et al. 1984; Ford and Pfefferbaum 1991; Friedman et al. 1993; Anderer et al. 1998), others do not confirm such an altered AEP pattern (Brown et al. 1983; Picton et al. 1984; Barrett et al. 1987).

This study aims to investigate age-related differences in the neural processing of spoken language during different modulations of the subject's selective attention. By comparing early AEP components (N1/P2 complex) between young adults (YA) and older adults (OA), we hypothesize to find task-related as well as age-related differences reflected as modulations of neurophysiological parameters (latency and amplitude). By using natural speech stimuli instead of the less complex sine-wave tones, CV syllables, or monosyllabic words, we aim to achieve a stronger generalization and comparability to real-life speech processing of our results.

## Material and Methods

### Participants

A total of 41 healthy, right-handed adults were measured: YA (*n* = 21, 11 women, *M* = 22.7 years; SD = 3.3) and older adults (*n* = 20, 10 women, *M* = 68.1 years; SD = 3.4). All participants were native German or Swiss German speakers and right handed according to the Annette Test for handedness (Annett 1970). All participants gave their informed written consent. The local ethical committee permitted the study.

### Stimulus material

Stimulus material consisted of 120 German words and 120 pseudowords. All words and pseudowords were disyllabic and corrected to a length of 800 msec using the Praat Software (Boersma 2002). Pseudowords were designed to respect rules of German phonotactics. The stimuli were spoken by a professional female speaker and recorded at a rate of 44.1 kHz. Additionally, two white noise stimuli of 500 and 1000 msec duration were generated. All stimuli were matched in intensity (amplitude normalization with the Praat Software). Stimulus material was presented using Presentation software, Version 14.9 (http//www.neurobs.com).

### Procedure

Before the EEG tasks commenced, participants were asked to complete behavioral tests assessing their speed of information processing (Kurztest für die Basisgrösse allgemeiner Intelligenz [KAI]; Lehr et al. 1991) and mental lexicon (Mehrfachwahl-Wortschatz-Intelligenztest [MWT-B]; Lehr 1977). Furthermore, older participants' hearing performance was controlled for (MAICO ST20; MAICO Diagnostics GmbH, Dortmund, Germany). This has been done to ensure the participant's appropriate hearing threshold. During the EEG experiment, participants were seated in a comfortable position about at a 1 m distance from a monitor in an electromagnetic and sound shielded booth. Stimulus material was presented via in-ear headphones (Sennheiser CX271, Sennheiser (Schweiz) AG, Unterengstringen, Switzerland) for two independent tasks, a “speech task” and a “nonspeech task.” To control for possible learning effects, 50% of the participants of each age group started with the speech task, the other half of the participants started with the nonspeech task, respectively. No explicit feedback was given during the experiment.

In the “speech task,” participants heard randomly presented words and pseudowords. Participants were instructed to decide if the previous heard stimulus was either a real word or a pseudoword.

**Figure 1:**
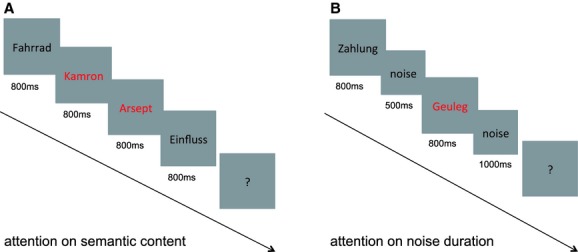
The two independent tasks of the study: (A) speech task, where real German words and pseudowords were presented; (B) nonspeech task, where white noise stimuli of two different duration were presented in addition to word and pseudowords. Red color indicates pseudoword whereas black color indicates correct German words. Participants were required to respond via button press at random time intervals indicated by a question mark on the screen.

The “nonspeech task” consisted of an additionally presented white noise stimulus as deviants between words and pseudowords. In this “nonspeech task,” the participants' task was to distinguish between the duration (either short or long) of the previously heard white noise stimulus. Participants were instructed to listen carefully to all of the presented stimuli. Additionally, participants were required to respond via button press at random time intervals indicated by a question mark on the screen (Fig. 1).

### EEG data acquisition

Electroencephalogram was recorded with a 128 channel system (EGI Eugene, OR), digitized at a sampling rate of 500 Hz, and band pass filtered between 0.3 and 100 Hz. Impedances were kept below 30 kΩ. Using Brain Vision Analyzer Software (Version 2.0.2, Brainproducts, Munich, Germany), data were referenced offline to linked mastoids and filtered between 1 and 15 Hz (48 dB/oct). Eye movements, eye blinks, or tonic muscle activity were removed using an independent component analysis (ICA) (Jung et al. 2000). Artifacts exceeding ±50 μV were automatically rejected and other artifacts were manually eliminated. The processed data were segmented, baseline corrected relative to the −100 to 0 msec prestimulus time, and averaged for each participant and stimulus type. In addition, grand means were averaged across all subjects for each age group separately. N1 was defined as the first negative deflection (latency window 100–150 msec) and P2 as the second positive deflection (latency window 160–300 msec). Statistical analysis was run over three midline electrodes (Fz, Cz, and Pz). Due to the lack of clear N1 and P2 waves at Fz and Pz, we only report results at the Cz electrode.

### Data analysis

#### Behavioral data

Independent sample *t*-tests were used to examine differences between the two age groups. We recorded the speed of information processing, assessed by the KAI, and also the verbal lexicon assessed by the MWT-B.

#### EEG data

We ran a 2 × 2 repeated measures analyses of variance (ANOVA) with task (speech and nonspeech) as the within-subject factors, and age (YA and OA) as the between-subject factors. ANOVAs were calculated separately for peak amplitude and latency of both the N1 and the P2 component. Furthermore, post hoc *t*-tests for independent samples were calculated for the amplitude and latency of the N1 and P2 component, as well as for task accuracy and response time (RT) between the age samples.

## Results

### Behavioral assessment

Age groups showed significant differences in their speed of information processing (*M*_OA_ = 26.455, SD = 9.68; *M*_YA_ = 21.45, SD = 2.067, *P *< 0.001) measured by means of the KAI and in their mental lexicon (*M*_OA_ = 126.15, SD = 12.06; *M*_YA_ = 109.00, SD = 13.405, *P *< 0.001) as measured by means of the MWT-B.

### EEG data

**Figure 2:**
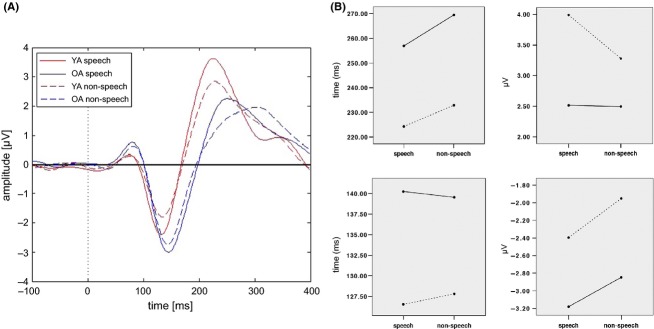
Grand means of the AEPs of both conditions and both age samples. (A) Speech task and nonspeech task AEPs for YA and OA. (B) Upper row: ANOVA plots for P2 latency (left) and P2 peak (right); Lower row: ANOVA plots for N1 latency (left) and N1 peak (right). YA are represented by dotted lines, whereas solid lines label OA.

Figure 2 shows the grand mean AEP of both age samples and conditions (A), as well as the ANOVA plots for N1 and P2 latency and peak values (B).

### Task accuracy

No significant differences were found between age groups in task accuracy. However, older participants showed significantly longer RT compared to young participants on the two tasks (*M*_OA_ = 2.94, SD_OA_ = 0.133, *M*_YA_ = 2.83, SD_YA_ = 0.099; *P *= 0.009; *P *< 0.001).

### N1

No statistically significant task-related differences in the N1 latency could be found in both age groups. However, post hoc *t*-test revealed that OA showed significantly longer latencies compared to YA on the speech task, (*P *< 0.001) as well as on the nonspeech task (*P* < 0.001).

Regarding the N1 amplitude, we found a main effect for *task* (*F*_2,41_ = 13.044, *P* < 0.001). A posteriori calculated *t*-tests showed a significantly stronger N1 amplitude in OA as compared to YA on the nonspeech task (*P* = 0.017). A similar trend could also be found on the speech task (*P* = 0.097). Focusing on task-related differences, we found stronger amplitude peaks in the speech task in comparison to the nonspeech task in YA (*P* = 0.002). A similar trend could be found in OA (*P* = 0.076).

**Figure 3:**
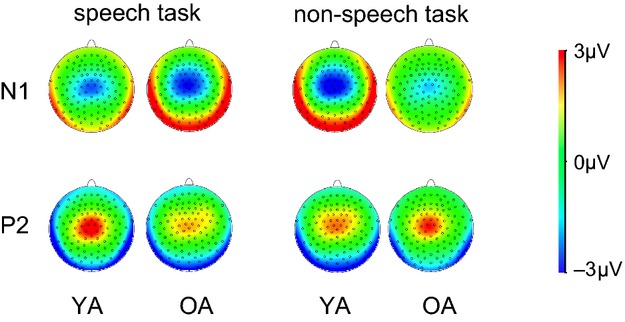
Mean topographical surface patterns of the examined AEP-components. Upper row: N1 component; Lower row: P2 component. Left cluster: speech task; right cluster: nonspeech task. In every cluster the left column represents YA, whereas the right column represents OA.

Topographical distribution (see Fig. 3) of the N1 component did not change with age: it exhibited a maximum over the Cz electrode in both samples.

### P2

Analyses of variance showed a main effect for *task* in the P2 latency (*F*_2,41_ = 14.418, *P* < 0.001) with prolonged latencies in the nonspeech compared with the speech task (*t*-tests in YA: *P* = 0.010; in OA: *P* = 0.021). Further analysis using independent sample *t*-tests revealed that OA showed significantly longer latencies compared to YA in the speech task (*P* < 0.001). This result also holds true for the nonspeech task (*P* < 0.001).

Regarding the P2 peak amplitude, we discovered a main effect for *task* (*F*_2,41_ = 5963, *P* = 0.019). Furthermore, we found an interaction effect for *age* × *task* (*F*_2,41_ = 5.326, *P* = 0.026) indicating an age-related modulation of the P2. Further analysis using independent sample *t*-tests showed enhanced amplitude in the YA as compared to OA in the speech task (*P* = 0.016), as well as a trend toward stronger peak amplitudes in the nonspeech task (*P* = 0.079). Interestingly, the P2 peak amplitude in older participants seems to be equal for both tasks, whereas young participants showed stronger P2 peaks in the speech task compared to the nonspeech task (*P* = 0.011). Because no difference in task accuracy between the two age groups could be found, this result indicates that modulation of the P2 component does not seem to be necessary for a successful task processing. Akin to the N1 topography no age-related effect in the topographical distribution of P2 was found (see Fig. 3).

## Discussion

In this AEP study we examined speech and nonspeech processing while two samples of young and senior volunteers performed both a speech and a nonspeech task. We found a general pattern of enhanced N1 amplitudes in YA compared to OA, whereas enlarged P2 amplitudes were found in OA compared to YA irrespective of the particular task demand. Additionally, we found a task-related modulation, namely, in the P2 component solely in YA: only YA showed stronger P2 amplitudes in the speech compared to the nonspeech task. The P2 component in OA revealed the same activation level, irrespective of the task. With respect to latencies, OA demonstrated generally longer latencies of the N1 and P2 components. We will discuss the implications of these results comprehensively in the following section.

### N1 and P2 latencies

Response latencies have been shown to reflect neural conduction time (Lister et al. 2011). Notably aging delays neural conduction and decreases neural precision (Iragui et al. 1993; Anderson et al. 2012; Kim et al. 2012). Therefore, longer N1 and P2 latencies in OA compared to YA may suggest age-related decrease in synchronous firing among the neural ensembles that generate N1 and P2 components (Walton et al. 1998, 2002; Walker et al. 2008). This finding implies that the auditory system in older adults is less able to precisely synchronize the neural activity to the onset of the speech stimuli, regardless of the focus of attention.

Assuming that some of the neuronal ensembles contributing to the generation of the N1 component overlap with those ensembles that elicit the P2 component, the prolonged latencies would represent a slower recovery process from the first, initial response, namely, the N1. Therefore, there might be an age-related difference in the refractory time exhibited by neurons in the auditory cortex of OA, leading to a longer recovery period before neurons are able to respond to a succeeding stimulus (Walton et al. 1998; Tremblay et al. 2003). This proposal receives further support by numerous studies that confirm an age-related decrease in speed of information processing in general, (Salthouse 1996, 2000) as well as for different cognitive functions, such as working memory (Sander et al. 2012) and divided attention (Park et al. 1989).

In addition to the measured differences in AEP latencies between YA and OA, a general attention-modulated pattern could be observed in both age samples. Both YA and OA showed prolonged latencies in the nonspeech compared to the speech task. The fact that this N1 and P2 latency pattern—representing an early level of auditory perception—is comparable in both age groups may indicate that the preliminary encoding of the stimuli is not affected by the aging process (i.e., aging of the auditory system and/or required cognitive functions). In contrast, the subsequent analysis of inflowing auditory information, as indexed by the P2 peak amplitude may be impoverished in older adults. Accordingly, Ostroff et al. (2003) suggest that precise encoding of sound duration declines after the fifth decade of life.

### N1 peak

We found a general pattern of stronger N1 amplitude in OA as compared to YA, regardless of their focus of attention. Additionally, we could measure enhanced N1 amplitude in the explicit speech task, as compared to the nonspeech task in both age groups.

N1 amplitude in humans marks the transition zone between perceptual processes partly driven by stimulus characteristics and partly affected by cognitive operations. It is often associated with cognitive functions such as stimulus encoding and the formation of a trace in the sensory memory (Näätänen and Picton 1987; Posner and Driver 1992). Explicitly focusing on specific characteristics of the paradigm, namely, speech stimuli, may lead to an increased neural responsiveness and therefore to stronger activation when processing the attended stimulus. The present observation of stronger N1 amplitudes in OA versus YA in the two tasks could be interpreted as a compensatory mechanism in the aging brain. By virtue of the recruitment of additional neurons, OA maintain their potential synchronous neural firing. The absence of an *age* × *attention* interaction indicates an attention-independent, general enhancement of potential involved neuronal ensembles. Thus, this mechanism may not be specifically attributed to stimulus encoding or processing of auditory speech and nonspeech material, but may also apply in other modalities. The recruitment of a wider activation pattern as a probable compensatory mechanism in OA has been documented to occur in other cognitive domains (Cabeza 2002).

However, Rao and colleagues associated the N1 component with task difficulty and task-related cognitive effort (Rao et al. 2010). Our findings fit with their interpretation, by revealing stronger N1 activity in speech stimuli as compared to nonspeech stimuli. Possibly, the differentiation between words and pseudowords requires more cognitive effort compared to distinguishing between noise stimuli of different durations because any presented speech stimuli must be matched with the participants' mental lexicon before a decision about its lexical status can be made. In contrast, it is obviously easier to decide about the duration of an acoustic stimulus, represented by only two possible options.

One may now wonder whether enhanced N1 amplitude in OA compared to YA can be interpreted as reflection of additional cognitive effort in OA. However, we assume it is more likely that a group-related difference in allocation of cognitive effort would occur at a later stage of stimulus processing and would thus be probably reflected by modulations of a late positivity.

### P2 peak

In this study, we measured enhanced P2 peak amplitude in YA compared to OA. Furthermore, whereas YA showed a task-related modulation of this component, no such modulation pattern could be observed in OA. The P2 component in OA rather seems to be uninfluenced by the focus of attention or by any characteristics of the presented stimuli.

P2 amplitude is usually associated with inhibitory processes and protection against interference from irrelevant stimuli (García-Larrea et al. 1992; Senderecka et al. 2012). According to García-Larrea and coworkers (1992), stronger inhibition leads to a stronger P2 amplitude. On the other hand, an age-related decline in inhibitory processes reflected by a decreased P2 component has been shown (Lister et al. 2011). Our findings argue against such an interpretation: YA showed a stronger P2 amplitude in the speech task versus the nonspeech task (i.e., in the task that requires less inhibition because no distractors have to be suppressed), whereas OA showed no modulation of the P2 component at all. Moreover, the topographic distributions of both AEP amplitudes at issue were comparable in both age groups. A shift into frontal regions, which is a typical indicator of inhibitory processes, was not observed in our study (see Fig. 3).

Two alternative explanations may account for the lack of any task-related modulation of the P2 component in OA. First, it could mean that the results are in line with the findings in YA, suggesting that P2 does not represent neural inhibition. Second, one may assume that an age-related decrease in the inhibition processes in older participants is already apparent in the AEP, but that this degeneration process is yet not implied by behavioral output. To flesh out these possibilities, a longitudinal assessment is necessary.

### Are N1 and P2 two independent substeps of sensory processing?

YA and OA showed similar task accuracy, but demonstrated substantial differences in age-related neurophysiological response pattern. Because N1 and P2 seem to be originated from (according to the topographical maps) distinct neural generators and processing steps, it can be assumed that the occurrence of both the N1 and P2 component is not an essential requirement for accomplishing the task.

In our view, two possible interpretations can be provided. The lack of an additional P2 task-related modulation in OA represents either:

1. An increased efficiency in processing speech stimuli. This, due to a longer exposure to language and speech that is also substantiated by an enhanced mental lexicon as measured with the behavioral MWT-B.
  Or
2. The consequence of an unspecific age-related neural degeneration process. In our opinion the latter argument seems more plausible because our stimulus material consisted of very frequent words. Its processing does not require a profound linguistic expertise.

The most important finding, however, of this study pertains to an inconsistency between behavioral and neurophysiological data. In particular, while we observed age-related differences in the neurophysiological pattern we did not find corresponding effects in the behavioral task accuracy (i.e., discrimination between words and pseudowords, or between short and long white noise stimuli, respectively).

Therefore, our findings indicate that the significantly different neural response patterns in younger and older participants were apparently not caused by an inability to understand or perform the tasks per se. This reasoning is in line with previous studies, who also found a difference between neural response patterns and behavioral responses (Woods 1992; Bellis et al. 2000).

## Conclusion

The present findings have several implications for the current understanding of the relationship between neural mechanisms and behavioral measurements during processing of spoken language at different stages of life. Psychophysical tasks require a conscious, behavioral response and may be affected by many internal or external factors, including selective attention, task demand, and general perceptual and motor skills. In contrast, ERPs are a complex multidimensional measurement of acoustic (or any other exogenous) events. AEPs comprise several parameters (amplitude, latency, polarity, and topography) that provide additional information compared to behavioral responses. A straightforward relationship between individual task performance and electrophysiology mirrored by behavioral measurement and the modulation of parameters of the N1/P2 complex can therefore not be taken for granted. The lack of consistency between behavioral and neurophysiological measurements may be attributed to the fact that various sensory and cognitive aspects of task performance that are reflected by distinct modulations of AEP parameters sum up in the behavioral response. This may result in an attenuation of the underlying complex interplay among age-, task-, and stimulus-related processes.
